# Retinoblastoma Binding Protein 2 (RBP2) Promotes HIF-1α–VEGF-Induced Angiogenesis of Non-Small Cell Lung Cancer via the Akt Pathway

**DOI:** 10.1371/journal.pone.0106032

**Published:** 2014-08-27

**Authors:** Lei Qi, Feng Zhu, Shu-hai Li, Li-bo Si, Li-kuan Hu, Hui Tian

**Affiliations:** 1 Department of Thoracic Surgery, Qi Lu Hospital, Shandong University, Jinan, Shandong Province, China; 2 Department of Thoracic Surgery, Shan dong Provincial Chest Hospital, Jinan, Shandong Province, China; Cincinnati Children’s Hospital Medical Center, United States of America

## Abstract

**Background:**

Pathological angiogenesis plays an essential role in tumor aggressiveness and leads to unfavorable prognosis. The aim of this study is to detect the potential role of Retinoblastoma binding protein 2 (RBP2) in the tumor angiogenesis of non-small cell lung cancer (NSCLC).

**Methods:**

Immunohistochemical staining was used to detect the expression of RBP2, hypoxia-inducible factor-1α (HIF-1α), vascular endothelial growth factor (VEGF) and CD34. Two pairs of siRNA sequences and pcDNA3-HA-RBP2 were used to down-regulate and up-regulate RBP2 expression in H1975 and SK-MES-1 cells. An endothelial cell tube formation assay, VEGF enzyme-linked immunosorbent assay, real-time PCR and western blotting were performed to detect the potential mechanisms mediated by RBP2 in tumor angiogenesis.

**Results:**

Of the 102 stage I NSCLC specimens analyzed, high RBP2 protein expression is closely associated with tumor size (P = 0.030), high HIF-1α expression (P = 0.028), high VEGF expression (P = 0.048), increased tumor angiogenesis (P = 0.033) and poor prognosis (P = 0.037); high MVD was associated with high HIF-1α expression (P = 0.034), high VEGF expression (P = 0.001) and poor prognosis (P = 0.040). Multivariate analysis indicated that RBP2 had an independent influence on the survival of patients with stage I NSCLC (P = 0.044). By modulating the expression of RBP2, our findings suggested that RBP2 protein depletion decreased HUVECs tube formation by down-regulating VEGF in a conditioned medium. RBP2 stimulated the up-regulation of VEGF, which was dependent on HIF-1α, and activated the HIF-1α via phosphatidylinositol 3-kinase (PI3K)/Akt signaling pathway. Moreover, VEGF increased the activation of Akt regulated by RBP2.

**Conclusions:**

The RBP2 protein may stimulate HIF-1α expression via the activation of the PI3K/Akt signaling pathway under normoxia and then stimulate VEGF expression. These findings indicate that RBP2 may play a critical role in tumor angiogenesis and serve as an attractive therapeutic target against tumor aggressiveness for early-stage NSCLC patients.

## Introduction

Non-small cell lung cancer (NSCLC) is among the most common malignancies leading to cancer-related death worldwide [1]. Despite numerous improvements in surgical techniques and adjuvant chemoradiotherapy for NSCLC over the last decades, the prognosis remains relatively poor [2]. A variety of new molecular markers and possible new targets have been found to treat the disease. However, tumor progression is a multistep process and the molecular mechanism underlying lung carcinogenesis is largely unclear.

Pathological angiogenesis is a relatively early event in carcinogenesis, and increased tumor angiogenesis is correlated with invasive tumor growth and metastasis and a poor prognosis [3], [4]. It has been proposed that vascular endothelial growth factor (VEGF) and hypoxia-inducible factor-1α (HIF-1α) play critical roles in tumor angiogenesis. VEGF, which is the most extensively characterized endothelial cell-specific angiogenic factor, leads to increased vascular permeability and plays a significant role in physiological and pathological angiogenesis [5], [6]. Accumulating evidence has demonstrated that HIF-1α, a heterodimeric protein composed of HIF-1α and HIF-1β subunits, is associated with various aspects of cellular and physiologic process. Under normoxia, HIF-1α is prolyl-hydroxylated, ubiquitylated and degraded in proteasomes by binding to the von Hippel Lindau (VHL) complex. Following hypoxia stabilization, HIF-1α binds to HIF-1β in the nucleus and initiates the transcription of target genes via the hypoxia-responsive element [7], [8], [9]. In recent years, many studies have suggested that HIF-1α also could lead to the elevated expression of various genes involved in diverse biological functions under normoxia, including cell proliferation, apoptosis, migration, invasion and angiogenesis [10], [11].

Retinoblastoma binding protein 2 (RBP2), a member of the JARID family of proteins, is a nuclear phosphoprotein with demethylase activity for lysine 4 of histone H3 (H3-K4) [12], [13], [14]. It appeared that RBP2 exerts its function partly by repressing the transcription of target genes involved in differentiation and that binding to retinoblastoma protein (pRB) converts RBP2 from a transcriptional repressor to a transcriptional activator [15], [16]. Recent research in lung cancer has established that RBP2 is correlated to tumor migration and invasion by directly binding to integrin β1 (ITGB1) promoters [17]. Another study demonstrated that RBP2 up-regulates the expression of N-cadherin and snail via the activation of Akt signaling [18]. Moreover, ITGB1 and Akt signaling are significantly correlated with tumor angiogenesis [19], [20], [21], [22]. Taken together, these results suggest an oncogenic role for RBP2 in tumor angiogenesis and progression.

In this study, RBP2 expression was found to be increased in NSCLC cell lines as well as in the NSCLC tissues from patients. To further investigate the potential roles of RBP2 in tumor angiogenesis, we provide evidence showing that high RBP2 expression in NSCLC cell lines significantly promotes tumor angiogenesis and elucidate the mechanism involved in the activation of Akt signaling, induction of HIF-1α protein accumulation and VEGF expression under normoxia.

## Materials and Methods

### Ethics Statement

This study was approved by the Ethics Committee of Qilu Hospital. Written informed consent was obtained from each patient to publish the case details, and the acquisition of tissue specimens was carried out as prescribed by the institutional guidelines.

### Patients

A total of 102 patients (71 men and 31 women, mean age 62±3.56 years) with stage I NSCLC who underwent complete tumor resection (lobectomy or pneumonectomy) with regional lymph node dissection between January 2006 and December 2008 at the department of Thoracic Surgery, Qilu Hospital, were included in the study. The histologic examination and grade of cancer cell differentiation were based on the classification system of the World Health Organization revised in 2004 and the TNM staging system of UICC 2009. In addition, 3 patients (2 cases with large cell cancer and 1 case with adenosquamous cancer) underwent complete tumor resection during this period were excluded owing to the too small sample size. The clinical characteristics of the 102 patients are presented in Table 1.

**Table 1:** Correlation of clinicopathologic variables with RBP2 protein and MVD in NSCLC.

		RBP2			MVD		
Category	No. of patients	high	low	*P* a	high	low	*P* a
Age				0.570			0.405
<60 years	42	20	22		21	21	
≥60 years	60	32	28		25	35	
Sex				0.933			0.197
Male	71	36	35		35	36	
Female	31	16	15		11	20	
Smoking history				0.712			0.172
Smoker	59	31	28		30	29	
Non- smoker	43	21	22		16	27	
Histology				0.846			0.564
SCC	52	27	25		22	30	
Adeno	50	25	25		24	26	
Differentiation				0.247			0.500
Well	14	10	4		5	9	
Moderate	60	28	32		26	34	
Poor	28	14	14		15	13	
T stage				0.030			0.064
T1	48	19	29		17	31	
T2	54	33	21		29	25	
HIF-1α				0.028			0.034
high	64	38	26		34	30	
low	38	14	24		12	26	
VEGF				0.048			0.001
high	59	35	24		35	24	
low	43	17	26		11	32	

a Chi-square test.

SCC squamous cell cancer,

Adeno adenocarcinoma.

### Immunohistochemistry

Immunohistochemical staining for RBP2, HIF-1α, VEGF and CD34 were carried out using the streptavidin-peroxidase method. In brief, 4-µm-thick sections were cut from paraffin-embedded blocks, and the slides were incubated with primary antibodies against RBP2 (Bethyl, Montgomery, TX, USA, dilution 1∶100), HIF-1α (BD Biosciences, Pharmingen, Lexington, MA, USA, dilution 1∶100), VEGF (A-20; Santa Cruz Biotechnologies, CA, USA, dilution 1∶150) and CD34 (sc-19621; Santa Cruz Biotechnologies, CA, USA, dilution 1∶100) overnight at 4°C. Subsequently, biotinylated secondary antibodies and peroxidase-conjugated streptavidin complex reagent were applied, followed by counterstaining with Mayer’s hematoxylin. Positive and negative controls were included in each step. Expression of the RBP2 protein was evaluated by calculating a total immunostaining score as the product of both the intensity score (0, negative staining; 1, weak staining; 2, moderate staining; 3, strong staining) and proportion score (0, none; 1, <10%; 2, 10–50%; 3, 51–80%; 4, >80%). Thus, the total score ranged from 0 to 7. The immunostained slides were evaluated by two independent investigators in a blinded fashion and reevaluated by these investigators under a multihead microscope in discordant cases to reach a consensus. For evaluation of the positive staining of RBP2, at least 3 sections or areas from each sample were scored.

For tumor-associated angiogenesis quantification, microvessel density (MVD) was evaluated by counting CD34-positive immunostained endothelial cells. CD34-positive endothelial cells as well as clusters of endothelial cells that were clearly separate from adjacent microvessels were measured as countable microvessels [23], [24], [25]. To quantify the MVD, five highly vascular areas were scanned at low power to identify “hot spots” and counted microscopically in high-power (200× magnification) fields. The average count of ten vision fields was recorded as the final MVD (two observers and five vascular hot spots each).

The cutoff value for RBP2 and MVD expression was determined based on a heterogeneity value measured through a log-rank statistical analysis with respect to overall survival [26]. The final staining score of 4 was chosen as the cutoff point for the discrimination between high and low RBP2 expression. Therefore, tumors with a final staining score ≥4 were defined as overexpressing the RBP2 protein. Tumors with microvessels ≥57 were classified as high MVD; tumors with microvessels <57 were classified as low MVD. HIF-1α was regarded overexpressed when >1% of nuclei were positive as described before [27]. Tumors with a final staining score ≥3 were defined as overexpressing the VEGF protein [28].

### Cell Lines, Culture Conditions, Transfection and Small Interfering RNA Treatment

The human lung adenocarcinoma cell lines A549, SPCA-1and H1975 were purchased from the National Cancer Institute (Bethesda, MD, USA). The human lung squamous cell line SK-MES-1 and the human bronchial epithelial cell line BEAS2B were obtained from American Type Culture Collection (Manassas, VA, USA). Human umbilical vein endothelial cells (HUVECs) were purchased from American Type Culture Collection (Manassas, VA, USA). The BEAS2B, A549, SPCA-1 and H1975 cells were cultured in Roswell’s Park Memorial Institute (RPMI) 1640 medium (Hyclone, Logan, USA) supplemented with 10% fetal bovine serum (FBS; Gibco, Gaithersburg, USA). The SK-MES-1 cells were grown in MEM (Gibco, Carlsbad, CA, USA) supplemented with 20% FBS. HUVECs were cultured in endothelial cell growth medium M199 supplemented with 15% FBS, 1 mg/ml low serum growth supplements and 2 mM glutamine. All cells were incubated in 5% CO_2_ at 37°C. RBP2 was overexpressed using pcDNA3-HA-RBP2, a generous gift from W.G Kaelin [12], and siRNA for RBP2 was purchased from Invitrogen (Carlsbad, CA, USA). The plasmid pcDNA3-HA-HIF-1α was purchased from Addgene (Plasmid 18949) [29], and siRNA for HIF-1α (ONTARGET plus SMART pool, L-004018) was purchased from Dharmacon RNA Technologies (Chicago, IL, USA). The plasmid pcDNA3 Myr HA Akt1 was purchased from Addgene (Plasmid 9008) [30]. For the small interfering RNA (siRNA) treatment, cells were incubated in 6-well plates (3.0×10^5^/well) overnight and then transfection was performed using Lipofectamine 2000 (Invitrogen, Carlsbad, CA, USA). The following siRNA sequences were used in this study: RBP2 siRNA1 5′-UUGUGUACUCGUCAAACUCUACUCC-3′; RBP2 siRNA2 5′-UUAACAUGCCGGUUAUCCAGGCUCU-3′; control siRNA 5′-UUCUCCGAAGGUGUCACGUTT -3′.

### Preparation of Conditioned Medium

The RBP2-siRNA1 H1975 cells, RBP2-siRNA2 H1975 cells and control-siRNA H1975 cells were cultured under serum-free conditions in RPMI 1640 medium for 24 h, respectively. The supernatant was then collected, centrifuged, filtered through a 0.22-mm filter (Millipore, Billerica, USA) and stored at −20°C until used in the enzyme-linked immunosorbent assay (ELISA) and tube formation assay.

### Endothelial Cell Tube Formation Assay

The tube formation assay was performed as described previously [31]. Briefly, HUVECs (1×10^4^/well) were seeded in 96-well plates coated with Matrigel (50 µl) and then incubated at 37°C for 1 h to polymerize. HUVECs (1×10^4^ cells) were seeded in wells with different conditioned media from RBP2-siRNA1 H1975 cells, RBP2-siRNA2 H1975 cells and control-siRNA H1975 cells. A VEGFR inhibitor (sunitinib malate, 2.5 µM) [32] was added to the conditioned medium of the control siRNA H1975 cells and recombinant human VEGF-165 (rhVEGF165, Millipore, Billerica, MA, USA, 2 ng/ml) was added to the conditioned medium of the RBP2-siRNA2 H1975 cells. The 96-well plates were incubated for 6 h, and tube formation was then photographed under an inverted microscope. The tube formation ability was quantified by counting the total number of complete tubes, and the average of three random ×200 fields per well was recorded as the value per well.

### VEGF Enzyme-Linked Immunosorbent Assay (ELISA)

The VEGF levels in the different conditioned media were determined using an ELISA kit (R&D Systems, Minneapolis, MN, USA) according to the manufacturer’s instructions and analyzed using a Labsystems Multiscan reader. The experiment was repeated twice with triplicate measurements in each experiment.

### SDS-PAGE and Western Blotting

Proteins were extracted using RIPA lysis buffer. Equal amounts (20 µg) of protein were subjected to SDS–PAGE analysis, transferred onto nitrocellulose membranes and probed with primary antibodies against RBP2 (Cell Signaling Technologies, Danvers, MA, USA), HIF-1α (Cell Signaling Technologies, Danvers, MA, USA), VEGF (Santa Cruz Biotechnologies, Santa Cruz, CA, USA), Akt and phospho-Akt (Ser-473) (Cell Signaling Technologies, Danvers, MA, USA). LY294002 was purchased from Beyotime Institute of Biotechnology (Haimen, China). Protein bands were detected using the enhanced chemiluminescence method (Millipore, Billerica, MA, USA). The optical band density was quantified (Imager of Alpha Corporation, San Leandro).

### RNA Extraction, Reverse-Transcription Polymerase Chain Reaction

Total cellular RNA in the cells from different treatments was extracted using Trizol (Invitrogen, Carlsbad, CA, USA). Complementary DNA (cDNA) was synthesized by SuperScript III First-Strand Synthesis System (Invitrogen, Carlsbad, CA, USA). Quantitative real-time PCR (QRT-PCR) was carried out using SYBR Green Supermix (Bio-Rad) for HIF-1α and VEGF according to the manufacturer’s instructions. The levels of HIF-1α and VEGF messenger RNA (mRNA) were normalized to the human β-actin expression level and calculated using the 2^(−ΔΔCT)^ method [33]. The primers for HIF-1α were 5′-TTTTTCAAGCAGTAGGAATTGGA-3′ (forward) and 5′-GTGATGTAGTAGCTGCATGATCG-3′ (reverse). The primers for VEGF were 5′-ATCTTCAAGCCATCCTGTGTGC- 3′ (forward) and 5′-CAAGGCCCACAGGGATTTTC-3′ (reverse). The primers for β-actin were 5′-GCATCCACGAAACTACCT-3′ (forward) and 5′-GAAAGGGTGTAACGCAAC-3′ (reverse). The cycling conditions were as follows: initial denaturation at 95°C for 30 s, followed by 40 cycles at 95°C for 5 s, 60°C for 30 s and 72°C for 15 s.

### Follow-up

All patients discharged from the hospital were followed-up at the outpatient clinic every 3 to 6 months. The follow-up evaluation of patients consisted of physical examination, blood tests, computed tomography, ultrasound examination, chest X-ray and fiberoptic bronchoscopy if necessary. Follow-up was completed in all patients until December 2013, and the median follow-up period was 66 months (range: 16∼96 months).

### Statistical Analyses

All statistical analyses were examined using SPSS 17.0 statistical software. Quantitative data were expressed as the mean ± SD for each group, and comparisons were performed using Student’s t-test. Chi-square tests were performed to examine the association between RBP2, MVD and various clinicopathologic factors. The correlation between intratumoral MVD and RBP2 protein levels was analyzed by a nonparametric test (Mann-Whitney U test). The follow-up time was censored if the patient was lost during follow-up. Survival curves were drawn using the Kaplan–Meier method and compared by the log-rank test. Multivariate Cox regression analysis was used to identify significant independent prognostic factors. *P* values were calculated from two-tailed statistical tests. A difference was considered statistically significant when *P*<0.05.

## Results

### 1. Correlation of RBP2 protein and MVD with clinicopathologic factors

**Figure 1:**
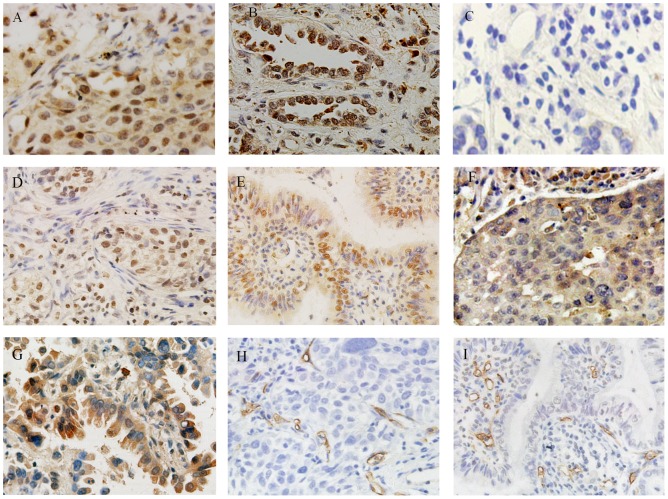
Immunohistochemical staining of the RBP2 protein and microvessels using the streptavidin–peroxidase method (magnification ×400). (A) High RBP2 protein expression in squamous cell cancer; (B) high RBP2 protein expression in adenocarcinoma; (C) negative RBP2 protein expression in NSCLC; (D) high HIF-1α expression in squamous cell cancer; (E) high HIF-1α protein expression in adenocarcinoma; (F) high VEGF expression in squamous cell cancer; (G) high VEGF in adenocarcinoma; (H) high CD34 expression in squamous cell cancer; (I) high CD34 expression in adenocarcinoma.

Immunohistochemistry with RBP2 (Fig. 1A and Fig. 1B) and HIF-1α (Fig. 1D and Fig. 1E) antibodies showed a positive reaction in the nucleus, and VEGF antibodies (Fig. 1F and Fig. 1G) showed a positive reaction in the cytoplasm of tumor cells. RBP2 was detected as being overexpressed in 52 (51%) of 102 NSCLC specimens according to the abovementioned criteria. Relationships between RBP2 protein expression and clinicopathologic factors were examined by the chi-square test and our data showed that RBP2 overexpression was associated with tumor size (*P* = 0.030), high HIF-1α expression (*P* = 0.028) and high VEGF expression (*P* = 0.048). However, there was no statistical significance in the relationships between RBP2 expression and other clinicopathologic variables (*P*>0.05, Table 1).

Intratumoral MVD was quantified by counting CD34-positive endothelial cells in the same series of lung cancer tissues (Fig. 1H and Fig. 1I), and the average number of microvessels in ten fields was counted as a measure of MVD for each sample. The average number of microvessels of MVD in each tumor sample ranged broadly, from 6.4 to 102. As described previously, tumors with microvessels ≥57 were classified as high MVD, and 46 cases (45.1%) showed high MVD. The chi-square test showed that high MVD was associated with high HIF-1α expression (*P* = 0.034) and high VEGF expression (*P* = 0.001), but no significant correlations were observed between MVD and other clinicopathologic factors (*P*>0.05, Table 1).

**Figure 2:**
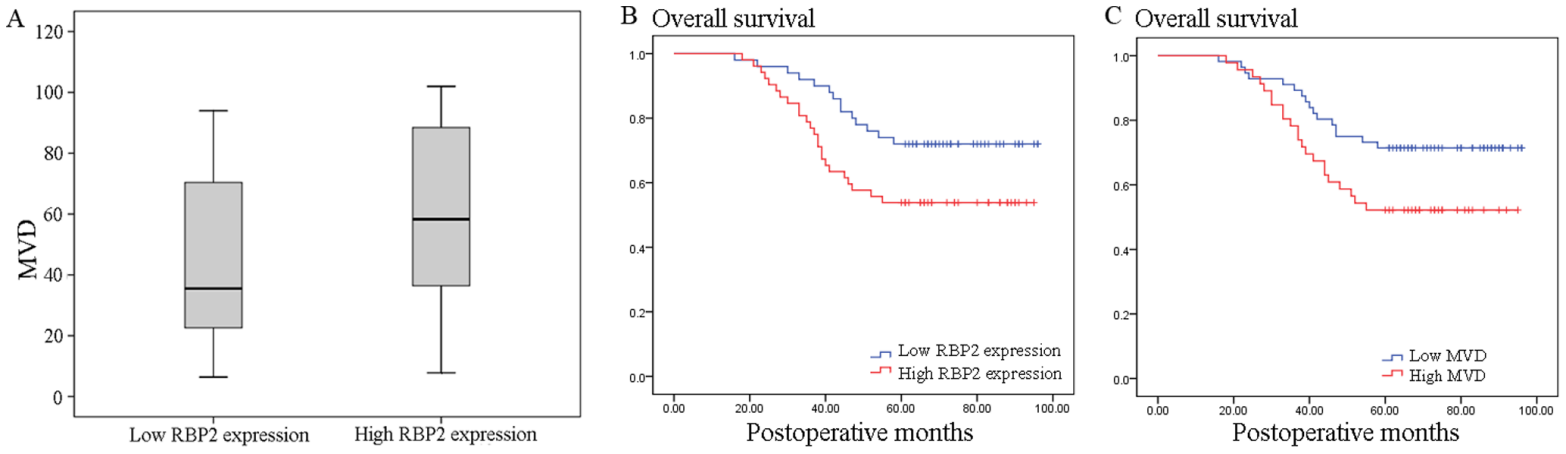
Correlation between RBP2 expression and MVD, and Kaplan–Meier curves of overall survival stratified according to RBP2 protein and MVD. (A) Correlation between RBP2 expression and MVD for stage I NSCLC. NSCLC with high RBP2 protein expression showed significantly higher intratumoral MVD than that with low RBP2 protein expression (*P* = 0.033, Mann-Whitney U test). (B) Kaplan–Meier curves of overall survival demonstrated a poor 5-year overall survival rate in patients with RBP2 protein overexpression (53.8% versus 72.0%, *P* = 0.037). (C) Kaplan–Meier curves of overall survival demonstrated a poor 5-year overall survival rate in patients with high MVD (52.2% versus 71.4%, *P* = 0.040).

Immunohistochemical staining of the serial sections of cancer tissues showed that the median MVD was 59.6 in the high RBP2 expression group (7.8 to 102) and 35.4 in the low RBP2 expression group (6.4 to 94). Furthermore, our statistical analysis demonstrated that high MVD was detected more frequently in tumors with RBP2 protein overexpression than in those without overexpression (*P* = 0.033, Mann-Whitney U test, Fig. 2A). These data suggest that RBP2 may promote pathological angiogenesis in NSCLC progression.

A Kaplan–Meier analysis of overall survival also demonstrated a poor 5-year overall survival rate in patients with RBP2 protein overexpression (53.8% versus 72.0%, *P* = 0.037; Fig. 2B) and high MVD (52.2% versus 71.4%, *P* = 0.040, Fig. 2C). All the statistically significant variables evaluated in the univariate analyses were included in a Cox proportional hazard regression model. The multivariate analysis indicated that only RBP2 had an independent influence on the survival of patients with stage I NSCLC (*P* = 0.044, Table 2).

**Table 2:** Univariate and multivariate analyses of prognostic variables.

	Univariate analysis	Multivariate analysis		
Variable	*P*	95% CI	RR	*P*
Sex	0.662	0.528–2.283	1098	0.802
Age	0.735	0.564–2.219	1.118	0.749
Histology	0.135	0.983–4.086	2.004	0.056
Differentiation	0.427	0.860–2.448	1.461	0.155
T stage	0.602	0.402–1.714	0.830	0.615
Smoking history	0.623	0.546–2.331	1.128	0.745
MVD	0.040	0.788–3.426	1.643	0.185
HIF-1α	0.306	0.236–1.606	0.616	0.322
VEGF	0.061	0.741–4.892	1.904	0.181
RBP2 protein	0.037	1.021–4.356	2.109	0.044

CI: confidence interval.

### 2. RBP2 is overexpressed in human NSCLC cell lines

**Figure 3:**
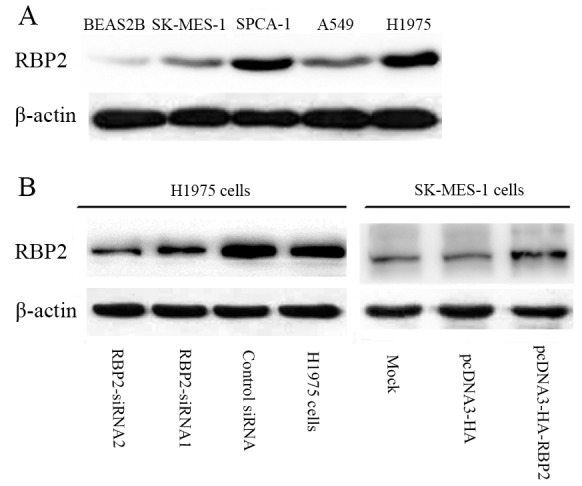
Expression of RBP2 in human NSCLC cell lines and the transfection efficiency of siRNA and pcDNA3-HA-RBP2. (A) RBP2 is overexpressed in human NSCLC cell lines SK-MES-1, A549, SPCA-1 and H1975 compared to the human bronchial epithelial cell line BEAS2B cells. (B) Effects of RBP2 siRNA1, RBP2 siRNA2 and pcDNA3-HA-RBP2 on the expression of the RBP2 protein.

The protein level of RBP2 was up-regulated in lung cancer cell lines SK-MES-1, A549, SPCA-1 and H1975 compared to the human bronchial epithelial cell line BEAS2B (Fig. 3A). The expression level of RBP2 in H1975 cells was higher than that in BEAS2B, SK-MES-1, SPCA-1 and A549 cells.

Two pieces of siRNAs and pcDNA3-HA-RBP2 targeting RBP2 were employed for a functional analysis in H1975 and SK-MES-1 cells. As shown in Fig. 3B, the results demonstrated that RBP2-siRNA1 and RBP2-siRNA2 could significantly down-regulate the expression of the RBP2 protein in H1975 cells. In addition, the two siRNAs showed nearly the same RNAi effects, whereas the negative control siRNA did not significantly affect the expression of RBP2. Meanwhile, pcDNA3-HA-RBP2 significantly up-regulated the expression of RBP2 in SK-MES-1 cells, whereas pcDNA3-HA did not significantly affect RBP2 expression. Therefore, RBP2-siRNA1, RBP2-siRNA2 and pcDNA3-HA-RBP2 were selected to further study the functions of the RBP2 protein in vitro.

### 3. Depletion of the RBP2 protein decreases HUVEC tube formation induced by conditioned medium

**Figure 4:**
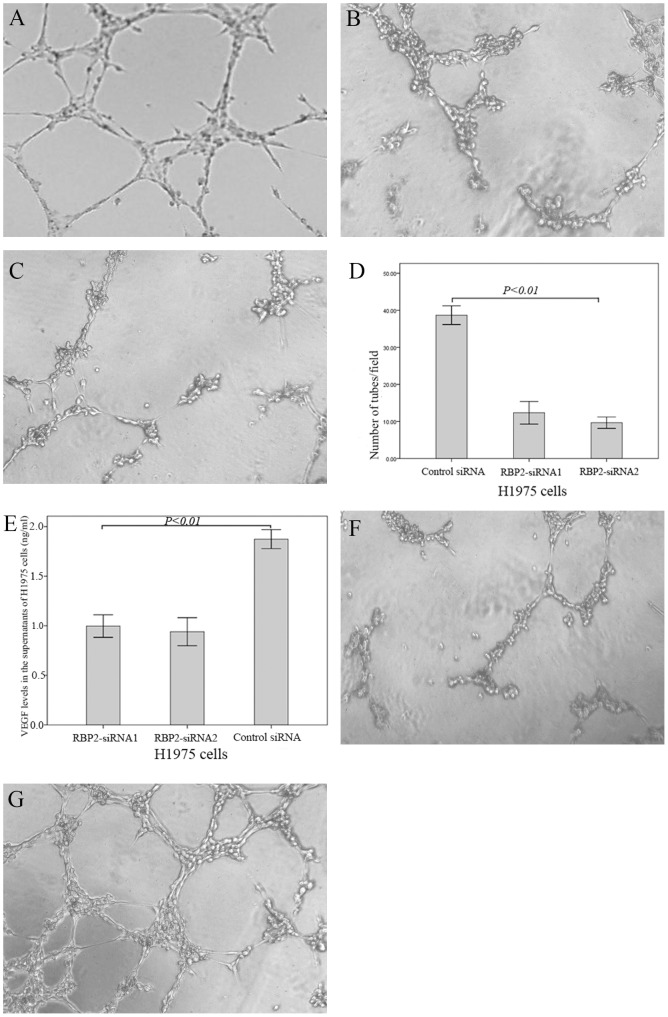
Down-regulation of the RBP2 protein decreased the tube formation by HUVECs induced by conditioned medium. Tube formation assay: (A) control-siRNA H1975 cells; (B) RBP2-siRNA1 H1975 cells; (C) RBP2-siRNA2 H1975 cells. (D) Quantitative analysis of the tube formation by HUVECs induced by conditioned medium; (E) Down-regulation of the RBP2 protein decreased the expression levels of VEGF in conditioned media. (F) The tube formation induced by the conditioned medium of control siRNA H1975 cells was blocked by the VEGFR inhibitor (sunitinib malate, 2.5 µM). (G) The reduced tube formation induced by the conditioned medium of RBP2-siRNA2 H1975 cells was rescued by adding VEGF-165 (2 ng/ml).

To evaluate the functional significance of RBP2 in tumor angiogenesis, RBP2 was knocked down in H1975 cell lines. The results demonstrated that the number of complete tubes induced by the conditioned medium of RBP2-siRNA1 H1975 cells (12.33+3.06) and RBP2-siRNA2 H1975 cells (9.67+1.53) was significantly reduced compared to that of the control siRNA H1975 cells (38.67+2.52, Fig. 4 A–D, *P*<0.01).

### 4. Down-regulation of RBP2 protein decreases the expression levels of VEGF in conditioned medium

Given the fact that high MVD was associated with high VEGF expression (chi-square test, *P* = 0.001, Table 1), we next explored the association between RBP2 and VEGF protein levels in conditioned media using an ELISA assay. The results demonstrated that the VEGF protein levels in the conditioned media of RBP2-siRNA1 (0.99±0.11 ng/ml) and RBP2-siRNA2 (0.94±0.14 ng/ml) H1975 cells were significantly lower than in the control siRNA H1975 cells (1.87±0.10 ng/ml), (Fig. 4E, *P*<0.01). In addition, our results suggested that the tube formation induced by the conditioned medium of the control siRNA H1975 cells was blocked with the addition of a VEGFR inhibitor (sunitinib malate, 2.5 µM, 10.33+2.22, *P*<0.01, Fig. 4F); the reduced tube formation induced by the conditioned medium of RBP2-siRNA2 H1975 cells was rescued by the addition of VEGF-165 (2 ng/ml, 41.03+3.25, *P*<0.01, Fig. 4G).

### 5. RBP2 stimulates HIF-1α and VEGF mRNA and protein expression

**Figure 5:**
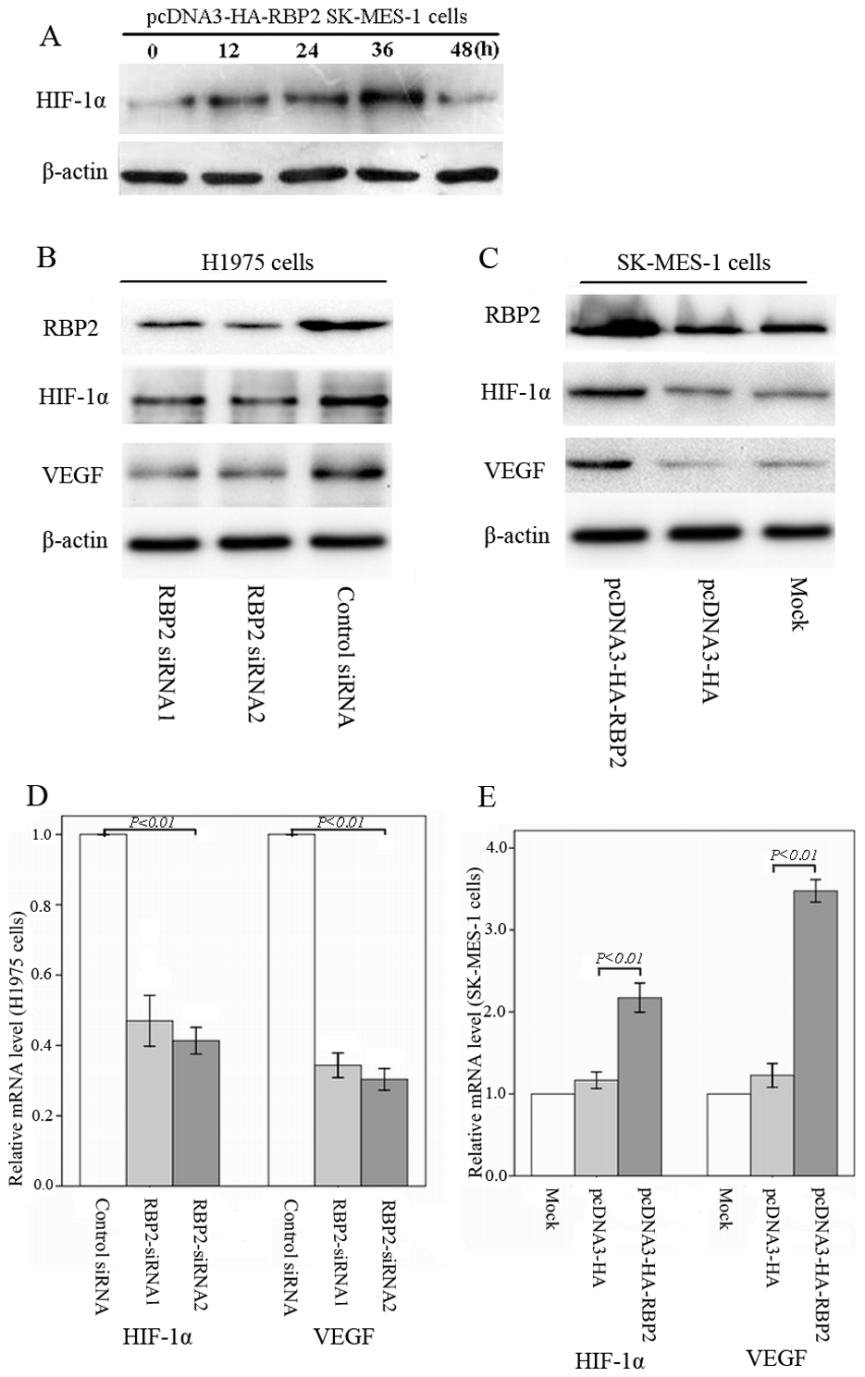
RBP2 stimulates the mRNA and protein expression of HIF-1α and VEGF. (A) RBP2 up-regulated the HIF-1α protein in a time-dependent manner under normoxic conditions. (B) Depletion of RBP2 decreased the expression of HIF-1α and VEGF in H1975 cells. (C) Up-regulation of RBP2 increased the expression of HIF-1α and VEGF in SK-MES-1 cells. (D) Real-time RT-PCR showed that the mRNA expression levels of HIF-1α and VEGF were significantly decreased in RBP2-depleted H1975 cells compared to control cells. (E) Real-time RT-PCR showed that the mRNA expression levels of HIF-1α and VEGF were significantly increased in RBP2-overexpressing SK-MES-1 cells.

HIF-1α is an important transcriptional factor and plays a crucial role in tumor angiogenesis. Moreover, the transcription factor HIF-1α regulates the expression level of various hypoxia-responsive genes, including VEGF [11]. We next investigated whether RBP2 could affect the expression of HIF-1α in ectopic RBP2-expressing SK-MES-1 cells. As shown in Fig. 5A, the enforced expression of RBP2 up-regulated the expression of the HIF-1α protein in a time-dependent manner under normoxic conditions, and the peak value of HIF-1α expression appeared at 36 hours after transfection was performed. However, HIF-1α was unstable and degraded by proteasomes under normoxia [9], and our results suggested that HIF-1α expression decreased after transfection was performed 48 hours. Therefore, to investigate the possible regulation of tumor angiogenesis by RBP2, we examined the expression of the transcription factors HIF-1α and VEGF in RBP2-overexpressing and -depleted NSCLC cells under normoxia at 36 hours after transfection. As shown in Fig. 5B and Fig. 5C, the down-regulation of RBP2 in H1975 cells led to the decreased expression of HIF-1α and VEGF, whereas ectopic RBP2 expression in SK-MES-1 cells by pcDNA3-HA-RBP2 led to the up-regulation of HIF-1α and VEGF.

The mRNA expression levels of HIF-1α (*P* ^siRNA1^ = 0.006, *P* ^siRNA2^ = 0.001) and VEGF (*P* ^siRNA1^ = 0.000, *P* ^siRNA2^ = 0.000) were significantly decreased in RBP2-depleted H1975 cells. Moreover, HIF-1α (*P* = 0.001) and VEGF (*P* = 0.000) were increased in RBP2-overexpressing SK-MES-1 cells compared to the control cells (Fig. 5D and Fig. 5E). These findings suggested that RBP2 plays an important role in the process of tumor angiogenesis through the up-regulation of HIF-1α and VEGF.

### 6. RBP2 induction of VEGF is dependent on HIF-1α

**Figure 6:**
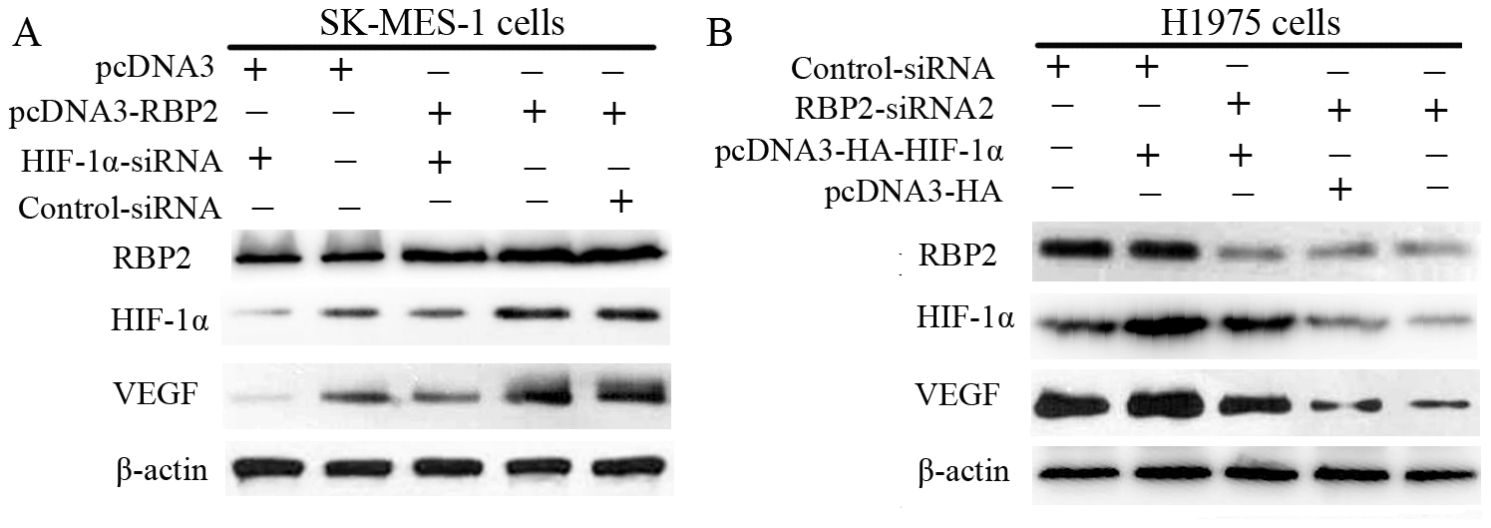
RBP2 induction of VEGF is dependent on HIF-1α. (A) Depletion of HIF-1α with an siRNA specific against HIF-1α in RBP2-overexpressing SK-MES-1 cells led to the down-regulation of VEGF compared with the scramble non-specific control siRNA. (B) Up-regulation of HIF-1α expression in RBP2-siRNA2 H1975 cells led to the up-regulation of VEGF.

To confirm the role of RBP2 in regulating HIF-1α in NSCLC cells, we modulated HIF-1α expression by transfecting cells with an siRNA specific against HIF-1α (si-HIF-1α) and a plasmid pcDNA3-HA-HIF-1α, and evaluated the expression of VEGF after 36 hours. As shown in Fig. 6A and Fig. 6B, knockdown of HIF-1α expression in ectopic RBP2-expressing SK-MES-1 cells led to the down-regulation of VEGF compared with the scramble non-specific control siRNA; up-regulation of HIF-1α expression in RBP2-depleted H1975 cells led to the up-regulation of VEGF. These results indicated that the RBP2-mediated tumor angiogenesis of NSCLC cells might partially be regulated through the activation of HIF-1α.

### 7. RBP2 activates HIF-1α via the PI3K/Akt signaling pathway

**Figure 7:**
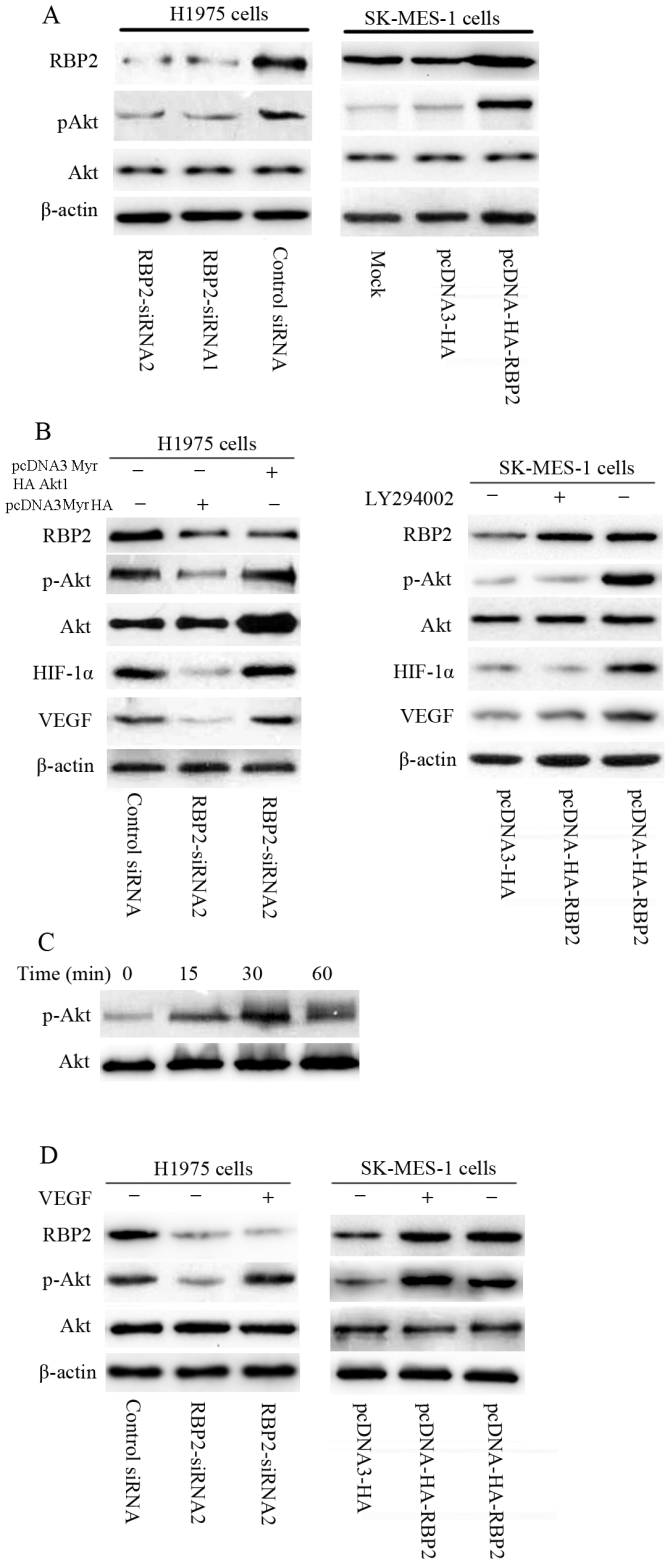
RBP2 activates HIF-1α via the PI3K/Akt signaling pathway. (A) Silencing RBP2 expression in H1975 cells significantly decreased the phosphorylation of Akt, and the forced expression of RBP2 in SK-MES-1 cells increased the activity of Akt. (B) When Akt was constitutively activated in RBP2-siRNA2 H1975, the expression of HIF-1α and VEGF were increased compared to the control. The PI3K/Akt inhibitor LY294002 significantly inhibited the expression of HIF-1α and VEGF in pcDNA3-HA-RBP2 SK-MES-1 cells. (C) Westernblots showing the time course of Akt phosphorylation in RBP2-siRNA2 H1975 cells due to VEGF-165 (25 ng/mL). (D) In the presence of recombinant human VEGF-165 stimulation, the activation of Akt was increased in RBP2-siRNA2 H1975 cells and RBP2-overexpressing SK-MES-1 cells (25 ng/mL, 30 minutes).

A recent study suggests that RBP2 regulates N-cadherin and snail through the activation of Akt signaling [18]. In addition, under normoxic conditions, the expression and activity of HIF-1α and the subsequent secreted angiogenic factors in cancer can be abnormally up-regulated by different signaling pathways [34], [35], [36] involving Akt and its downstream effectors [20], [22]. Therefore, we hypothesized that RBP2 regulates HIF-1α through the activation of Akt signaling, and we further sought to detect the signaling mechanisms involved in RBP2-mediated tumor angiogenesis. Our results revealed that silencing RBP2 expression with either RBP2-siRNA1 or RBP2-siRNA2 in H1975 cells significantly decreased the phosphorylation of Akt, whereas the forced expression of RBP2 with pcDNA3-HA-RBP2 in SK-MES-1 cells increased the activity of Akt (Fig. 7A). Moreover, when a constitutively active form of Akt in RBP2-siRNA2 H1975 cells was expressed, the expression of HIF-1α and VEGF were increased compared to the control; the PI3K/Akt inhibitor LY294002 significantly inhibited the expression of HIF-1α and VEGF in pcDNA3-HA-RBP2 SK-MES-1 cells (Fig. 7B). These data suggest that RBP2 promotes tumor angiogenesis through the activation of the PI3K/Akt signaling pathway in NSCLC cell lines.

VEGF has been shown to be a potent activator of Akt in some cases [37]. We next explored whether VEGF could activate Akt. As shown in Fig. 7C, treatment of RBP2-siRNA2 H1975 cells with recombinant human VEGF-165 (25 ng/mL) increased the activation of Akt within 15 to 30 minutes. Therefore, to investigate the possible regulation of Akt by VEGF, we examined the expression of p-Akt at 30 minutes after recombinant human VEGF-165 was added. As shown in Fig. 7D, in the presence of recombinant human VEGF-165 stimulation, the activation of Akt was increased in RBP2-depleted H1975 cells and RBP2-overexpressing SK-MES-1 cells (25 ng/mL, 30 minutes). Thus, our results suggested that VEGF indeed increases the activation of Akt, as regulated by RBP2.

## Discussion

RBP2, a member of the JARID family, was originally identified as a tumor suppressor retinoblastoma protein (pRB) binding partner that possesses H3-K4 demethylase activity [12], [13], [14]. Previous studies demonstrated that the oncogenic protein RBP2 was overexpressed in gastric cancer and NSCLC, which correlated with tumor senescence, proliferation, migration and invasion [17], [38]. However, the present study is the first study to investigate the role of the RBP2 protein in relation to angiogenesis of in NSCLC patients.

The data of our study showed that high RBP2 expression is common in stage I NSCLC tissues and is significantly associated with tumor size, high HIF-1α expression, high VEGF expression and a poor prognosis, suggesting that the RBP2 protein is involved in the aggressive progression of NSCLC. Pathological angiogenesis plays an essential role in tumor initiation, progression and metastasis and also has prognostic importance in various types of human solid tumors [39], [40]. Tumor angiogenesis was evaluated by CD34-determined intratumoral MVD in the present study, and high MVD was also associated with high HIF-1α expression and high VEGF expression. Moreover, Mann-Whitney U test showed that high RBP2 expression was correlated with increased MVD in patients with stage I NSCLC, demonstrating a novel angiogenic role for RBP2 in NSCLC invasiveness and metastasis. Thus, the RBP2 protein may promote pathological angiogenesis through the up-regulation of HIF-1α and VEGF in NSCLC progression.

NSCLC is an angiogenesis-dependent tumor, and angiogenesis plays pivotal roles in progression and blood-borne metastases [41], [42]. The pathological angiogenesis of tumors is a complex, multistep process involving various cytokines [43], [44]. The possible angiogenic potential of the RBP2 protein in vitro was analyzed by the tube formation assay. Our results showed that down-regulation of the RBP2 protein could significantly decrease HUVEC tube formation induced by conditioned medium. VEGF is a key pro-angiogenic effector and plays a significant role in physiological and pathological angiogenesis [5], [6], and we further detected the VEGF levels in different conditioned media. The results of our ELISA assay demonstrated that the VEGF protein levels in the conditioned medium of RBP2-siRNA H1975 cells were significantly lower than that of the control siRNA H1975 cells. In addition, the tube formation induced by the conditioned medium of control-siRNA H1975 cells was blocked by sunitinib malate which was a VEGFR inhibitor and the reduced tube formation induced by the conditioned medium of RBP2-siRNA2 H1975 cells was rescued by adding VEGF-165. These findings indicated that the tube formation induced by RBP2 might be VEGF dependent.

Transcription factor HIF-1α plays a crucial role in tumor angiogenesis and regulates the expression level of VEGF [11]. Many studies have suggested the importance of increased HIF-1α levels in the tumorigenesis and progression of various cancers by promoting tumor angiogenesis and the development of other hallmarks of cancer [45]. In addition, previous studies revealed that the expression of HIF-1α and VEGF is up-regulated in NSCLC and is related to a poor prognosis and worse overall survival [46], [47], [48]. We next explored the relationship between RBP2 and HIF-1α and VEGF protein expression in SK-MES-1 and H1975 cells. Our results suggested that the up-regulation of RBP2 leads to increased expression of HIF-1α and VEGF at both the mRNA and protein levels; in contrast, the depletion of RBP2 resulted in decreased expression levels of HIF-1α and VEGF. Interestingly, with RBP2 overexpression, the increased expression of VEGF induced by RBP2 was blocked by HIF-1α siRNA. Moreover, the enforced expression of HIF-1α in RBP2-depleted H1975 cells led to the up-regulation of VEGF. Therefore, our results confirmed that RBP2 is a non-hypoxic inducer of HIF-1α expression that modulates the process of tumor angiogenesis via HIF-1α-VEGF signaling. Targeting RBP2 signaling by novel approaches would be useful for reversing tumor angiogenesis.

The phosphatidylinositol 3-kinase (PI3K)/Akt signaling pathway plays a pivotal role in the core of the molecular signaling net work that governs proliferation, apoptosis, invasion and migration in many cell types [49], [50]. Our observations showed that RBP2 increased the expression levels of HIF-1α and VEGF via the activation of PI3K/Akt signaling pathway. However, as VEGF has been shown to be a potent activator of Akt in some cases [37], we next explored the possible regulation of Akt by VEGF. Recombinant human VEGF-165 could stimulate the phosphorylation of Akt in RBP2-depleted H1975 cells and RBP2-overexpressing SK-MES-1 cells. As stated above, our results suggested that VEGF and Akt may be involved in a feedback loop.

Integrins, a group of glycoprotein receptors, mediate cell adhesion and interaction with the extracellular matrix (ECM). In many cell types, integrins and other membrane receptors form macromolecular complexes, constituting signaling platforms at the adhesion sites. The Akt signaling pathway can be triggered by integrinβ1-mediated adhesion through Fak, which binds to the p85 subunit of PI3K, or via the Src–vinculin complex [51], [52], [53], [54]. Interestingly, RBP2 was found to act as a transcription activator for integrinβ1 (ITGB1) by directly binding to its promoter, suggesting that ITGB1 is a direct and specific downstream target of RBP2 [17]. As stated above, the transcription of integrinβ1 activated by RBP2 may facilitate the activation of the Akt signaling pathway and play a critical role in RBP2-mediated tumor angiogenesis.

However, the activity, expression, synthesis and stability of HIF-1α are extremely complex, involving various factors such as MEK, PI3K, mTOR, eIF-4E, p70S6K, RACK1 and Hsp90 [55], [56], [57]. RBP2 plays a dual role by acting as a transcriptional repressor and transcriptional activator [15], [16] and may have an affect on the activity, expression, synthesis and stability of HIF-1α. Therefore the precise mechanisms necessary for the RBP2 protein to induce HIF-1α still need to be further researched.

In conclusion, we provided evidence showing that high RBP2 expression and high MVD were common in stage I NSCLC tissues and closely associated with poor prognosis. In addition, high RBP2 expression was closely associated with tumor size, high HIF-1α expression, high VEGF expression and increased tumor angiogenesis. Multivariate analysis indicated that RBP2 had an independent influence on the survival of patients with stage I NSCLC. The RBP2 protein may play a critical role in NSCLC tumor angiogenesis by enhancing HIF-1α and VEGF expression under normoxia via the PI3K/Akt signaling pathway. Moreover, VEGF could increase the activation of Akt regulated by RBP2. These findings indicate that RBP2 could serve as an attractive therapeutic target against angiogenesis for early-stage NSCLC patients.
